# Individual Differences in How Desirable People Think They Are as a Mate

**DOI:** 10.1007/s10508-023-02601-x

**Published:** 2023-05-08

**Authors:** Zsófia Csajbók, Zuzana Štěrbová, Gayle Brewer, Cristina A. Cândea, Charlotte J. S. De Backer, Ana Maria Fernández, Maryanne L. Fisher, Justin R. Garcia, Daniel J. Kruger, Karlijn Massar, Elisabeth Oberzaucher, Katinka J. P. Quintelier, Renske E. van Geffen, Jaroslava Varella Valentova, Marco Antonio Correa Varella, Peter K. Jonason

**Affiliations:** 1 Faculty of Humanities, Charles University, Prague, Czech Republic; 2 Faculty of Arts, Charles University, Prague, Czech Republic; 3 Institute of Population Health, University of Liverpool, Liverpool, UK; 4 Faculty of Biology, University of Bucharest, Bucharest, Romania; 5 Department of Communication Sciences, University of Antwerp, Antwerp, Belgium; 6 Faculty of Humanities, University of Santiago of Chile, Santiago, Chile; 7 Department of Psychology, Saint Mary’s University, Halifax, Canada; 8 Kinsey Institute, Indiana University, Bloomington, IN USA; 9 Institute for Social Research, University of Michigan, Ann Arbor, MI USA; 10 Department of Work and Social Psychology, Maastricht University, Maastricht, Netherlands; 11 Department of Evolutionary Anthropology, University of Vienna, Vienna, Austria; 12 School of Business and Economics, Management and Organisation, Vrije Universiteit Amsterdam, Amsterdam, Netherlands; 13 Amsterdam University of Applied Sciences, Amsterdam, Netherlands; 14 Department of Experimental Psychology, University of Sao Paulo, Sao Paulo, Brazil; 15 Department of General Psychology, University of Padua, Via Venezia, 12, 35131 Padua, PD Italy; 16 Institute of Psychology, Cardinal Stefan Wyszyński University, Warsaw, Poland

**Keywords:** Mate value, Mate desirability, Attractiveness, Dark Triad, Sex differences, Mating

## Abstract

Mate value is an important concept in mate choice research although its operationalization and understanding are limited. Here, we reviewed and evaluated previously established conceptual and methodological approaches measuring mate value and presented original research using individual differences in how people view themselves as a face-valid proxy for mate value in long- and short-term contexts. In data from 41 nations (*N* = 3895, *M*_age_ = 24.71, 63% women, 47% single), we tested sex, age, and relationship status effects on self-perceived mate desirability, along with individual differences in the Dark Triad traits, life history strategies, peer-based comparison of desirability, and self-reported mating success. Both sexes indicated more short-term than long-term mate desirability; however, men reported more long-term mate desirability than women, whereas women reported more short-term mate desirability than men. Further, individuals who were in a committed relationship felt more desirable than those who were not. Concerning the cross-sectional stability of mate desirability across the lifespan, in men, short- and long-term desirability rose to the age of 40 and 50, respectively, and decreased afterward. In women, short-term desirability rose to the age of 38 and decreased afterward, whereas long-term desirability remained stable over time. Our results suggest that measuring long- and short-term self-perceived mate desirability reveals predictable correlates.

**Supplementary Information:**

The online version contains supplementary material available at 10.1007/s10508-023-02601-x.

## Introduction

Mate value, or the overall assessment of an individual’s desirability as a romantic or sexual partner, is thought to be one of the most important driving forces of mate choice (Buss & Schmitt, 2019; Sela et al., 2017). It predicts higher mating standards, more freedom to choose partners, and a greater tendency to reject others (Conroy-Beam, 2017; Conroy-Beam et al., 2019a; Csajbók & Berkics, 2022; Csajbók et al., 2019, 2023; Jonason et al., 2015; Wenzel & Emerson, 2009). However, popular and useful it has been for mating research, its conceptualization and thus its operationalization tends to be unsystematic, contradictory, and limited (for a summary, see Table 1). For instance, researchers have focused on self-evaluations of traits that may be subject to impression management, failed to include both long-term and short-term mating contexts, or focused on objective features that are particularly hard to assess and may not reflect the gestalt of desirability as a mate.

**Table 1:** Methodological approaches to measuring mate value

Approach	Study	Measures	Length	Sample items
General (self-perceived)	Edlund & Sagarin, 2014	The mate value scale	4 items	“Overall, how would you rate your level of desirability as a partner on the following scale?” “Overall, how would members of the opposite sex rate your level of desirability as a partner on the following scale?”
	Landolt et al., 1995	Self-perceived mating success scale	8 items	“Members of the opposite sex that I like, tend to like me back.” “I can have as many sexual partners as I choose.” “Members of the opposite sex are attracted to me.”
	Sela et al., 2017	Self-perceived short-term mate value	1 item for self	“How desirable are you for a short-term uncommitted relationship (e.g., a one-night stand)?”
Combined: dimensional and general (self-perceived)	Brase & Guy, 2004	Single question of desirability with description of factors	1 item	“Many people look at specific characteristics in choosing their potential marriage partners. Some common desirable traits include: Being socially exciting, age, being physically attractive, having a good sense of humor, having good financial/professional status, being of high intelligence, being in good health, and liking children. Overall, how would you rate your level of desirability as a partner (p. 476)”
	Jonason et al., 2019, 2020	Li’s Mate Value scale	20 items loading on 3 factors	“My physical attractiveness is the main reason why people are romantically interested in me.” “My friends are proud to recommend me to others as a long-term relationship partner.” “Getting a desirable girlfriend/boyfriend seems hopeless to me.” Factors: Short- and long-term mating attractiveness, general undesirability
	Conroy-Beam et al., 2019a	Euclidean distance of self from average mate preferences	5 items	Intelligence, kindness, health, physical attractiveness, and financial prospects
	Csajbók et al., 2023 (the current study)	Short- and long-term mate desirability	4 items	Short-term desirability: “If you were single, how easy would it be for you to find a short-term mate for romance?” “If you were single, how easy would it be for you to find a short-term mate for only sex?” long-term desirability: “If you were single, how easy would it be for you to find a potential long-term mate?” “If you were single, how easy would it be for you to find a long-term relationship potentially leading to marriage?”
Dimensional (self-perceived)	Fisher et al., 2008	Components of Mate Value Survey	22 items loading on 7 factors	“Members of the opposite sex are attracted to me.” “I have a large network of friends” “I would make a good parent” “I have a tendency to display my wealth” “I would like members of the opposite sex to consider me physically attractive” “After I date someone they often want to date me again” “I often worry about not having a date” Factors: Views of the opposite sex, Sociability, Parenting, Wealth, Looks, Relationship history, Fear of failure
	Kirsner et al., 2003	Mate Value Inventory	17 items, the ratings are summed up to an overall value	ambitious, attractive face, attractive body, desires children, emotionally stable, enthusiastic about sex, faithful to partner, financially secure, generous, good sense of humor, healthy, independent, intelligent, kind and understanding, loyal, responsible, and sociable
	e.g., Csajbók & Berkics, 2017; Goodwin et al., 2012; Regan, 1998	Using the same factors for ideal partner and self-evaluation	Depends on the included *N* of factors	Csajbók & Berkics, 2017: Warmth, Attractiveness, Status, Intellect, Stability, Passion, Dominance; Goodwin et al., 2012: Caring, Socially attractive, Passionate romantic, Adventurer, Mature confident; Regan, 1998: Interpersonal skill and responsiveness, Intellect, Physical attractiveness, Social status, Interpersonal power, Family orientation
By a proxy (objective or other-perceived)	Singh, 2002	Waist-to-hip ratio	(short)	
	Fisher et al., 2008	*N* of past partners	(short)	
	Feinberg, 2008	Voice and facial features	(can be costly)	
	Pflüger et al., 2012	*N* of children during their lifespan	(short, but can be costly)	
	Buss & Shackelford, 2008; Clark, 2004; Montoya, 2008	Physical attractiveness rated by independent raters	(can be costly)	

### Mating Market Operations

Largely the idea of mate value comes from the application of supply-and-demand dynamics to the context of romantic and sexual relationships, which, like the market of used cars, should be the result of the evaluation of features of product, the buyer, and the context (Bongard et al., 2019; Noë & Hammerstein, 1995; Pereira et al., 2020; Valentova et al., 2016; Walter et al., 2020). Each car has specific conditions, both objective and subjective. The brand, model, and age of the car are easily quantified, and maybe more objective than the condition of the interior, the unknown effects of a car accident in the past, or its general maintenance. These features are subjective, because it is subject to momentary needs, such as how fast somebody wants to find a car (or a partner), what specific offers and desires somebody has, all of which change as people age and experience new circumstances (e.g., having a baby and looking for a mate on the secondary mating market; Potarca et al., 2017). Eventually, the price of something will be exactly known only at the time of the purchase, and it will be applicable only to that exact moment between that exact buyer and seller. The exact value of a used car, like potential mates, is dynamic, developing and updating over time (e.g., seeing that another is chosen by others increases the value assigned to them; Deng & Zheng, 2015), making its measurement particularly tricky.

In addition, what someone is willing to pay (i.e., other-perceived mate value) is distinct from how valuable people see themselves further complicating matters and is subject to fluctuations in how much potentially objective qualities like waist-to-hip ratio, masculinity, and voice-pitch are valued by someone (Arnocky, 2018; Csajbók et al., 2019; Edlund & Sagarin, 2014; Feinberg, 2008; Fisher et al., 2008; Lidborg et al., 2022; Pereira et al., 2019; Singh, 2002; Valentova et al., 2019). Although researchers would like to approximate the objective value of somebody on the mating market, it cannot be measured because of the differences in the so-called eye of the beholder. It is all circumstantial. The transactions of mate choice, the offers and rejections are difficult to trace and map. Individuals infer rejection or commence flirting based on minimal information obtained from subtle interactions, even multiple times per day. To overcome these problems, we support a simple solution: ask individuals how desirable they think they are, as a mate, in others’ eyes (Edlund & Sagarin, 2014).

The most fundamental assumption of the utility of mate value, and mating market operations in general, is that people have a notion of their own which drives their behavior even if they cannot identify its underlying formula or processes (Brase & Guy, 2004; Edlund & Sagarin, 2014). Self-perceived mate value is the self-evaluation that the individual appreciates as their own mating potential. It should mirror their own ability to find a partner should they become single. In other words, it should reflect how many people are interested in initiating a relationship with them (Edlund & Sagarin, 2014; Fisher et al., 2008). It does not mean that the person would accept every offer for sex or a date, but the magnitude of the interest to initiate a relationship with them should be an indicator of their mate value.

Self-perceived mate value is likely to operate like self-esteem in the sociometer model (Csajbók et al., 2019; Kavanagh et al., 2010; Leary & Baumeister, 2000; Li et al., 2013). Although self-esteem and self-perceived mate value are conceptually different, they have similar characteristics; both are sensitive to rejections and social comparisons (Campbell & Wilbur, 2009; Pass et al., 2010; Zhang et al., 2015). Self-perceived mate value is associated with the person’s self-esteem, developmental history, and even psychopathology, for example depression (e.g., Brase & Dillon, 2022; Kirkpatrick et al., 2002; Kirsner et al., 2003). Humans’ long adolescence offers the opportunity to practice and learn the principal aspects of mate choice. They can learn, for example, their own mate value through the series of mating offers and rejections they experience during this time (Fletcher et al., 1999; Miller & Todd, 1998). This learning period is also the stage for the individuals to set their aspiration levels, or ideal expectations based on what they may be likely to achieve (Miller & Todd, 1998). Perhaps the most iconic real-life simulation of the process of individuals learning their self-perceived mate value, as well as the matching phenomenon, was conducted by Ellis and Kelley (1999). In this experiment, participants in a classroom received a randomly assigned number on their forehead. Without knowing their own number, trying to couple with the best possible number in the room, the participants coupled up similar with similar. Interestingly, at the end, the participants could guess their own number well. This is showcasing how individuals learn about their own mate value based on subtle nonverbal signaling even in these neutral and simplified circumstances.

### Measurement of Mate Value

As the conceptualization of mate value had some difficulties, its measurement faced some obstacles as well (Edlund & Sagarin, 2014). So far, self-perceived mate value was measured by separate factors, or holistically, by one overall/general construct (e.g., Fisher et al., 2008; Edlund & Sagarin, respectively; Table 1). Even though objective mate value may not exist at all, not every researcher agrees. Some used reproductive success as a proxy of objective mate value (Pflüger et al., 2012) whereas others employed independent raters to assess the physical attractiveness of the participants, although one could argue that such measure is also subjective from the raters’ perspective rather than objective (e.g., Buss & Shackelford, 2008; Montoya, 2008). The variation in measurement approaches demonstrates the general disagreement in what mate value is conceptually.

The question of what is a reliable mate value measure evokes the same problem around the concepts of objective versus self-perceived and other-perceived mate value. Even if we accept the possibility of an objective mate value, attempts to measure objective mate value indicators can be costly and impractical. For example, asking several independent raters to provide an estimation of a target’s mate value after a lengthy face-to-face interview; or recording the individuals’ number of children once their reproductive age was over would require substantial work with uncertain outcomes. Mating behavior, after all, was better predicted by what people thought of themselves than what others saw in them (Arnocky, 2018), thus a costly measure of objective mate value may not be worth the efforts. To overcome these problems, Edlund and Sagarin (2014) suggest to simply asking how much the participants are worth in the others’ eyes.

### Correlates of Mate Value

Research relying on an evolutionary psychological perspective has been useful in identifying some of the factors influencing self-perceived mate value or desirability. Factors like warmth, trustworthiness, and intelligence are important indicators of good parenthood and partnership for both men and women (Fletcher et al., 1999). Similarly, physical attractiveness and access to resources are among the most important indicators of mate quality and cross-culturally desired in women and men, respectively (Buss, 1989; Buss & Schmitt, 1993; Fletcher et al., 1999; Walter et al., 2020). Thus, objective features predicting attractiveness and status should correlate with mate desirability. However, the relationship is not that straightforward because, for example, while aging may correlate with career progress in the case of men, thus indicating higher status, women gradually lose their physical attractiveness over time, but tests of this idea are equivocal at best (Arnocky, 2018; Brase & Guy, 2004; Csajbók & Berkics, 2017; Csajbók et al., 2019; Fernandez et al., 2014; Mafra & Lopes, 2014). We should thus explore in more detail, preferably with non-linear methods, how self-perceived mate desirability is associated with age in men and women, considering that mate value’s strongest predictor is self-, but not other-perceived attractiveness (Clark, 2004; Csajbók & Berkics, 2017). Moreover, being in a relationship (as compared to being single) may be associated with higher self-perceived mate desirability because (1) mating success should act as feedback to a person that they are of value or (2) those with higher desirability should be more likely to be chosen for relationships and more willing to reject others (i.e., being choosier; Regan, 1998).

Another theory used to understand mate value is life history theory (Del Giudice et al., 2016; Figueredo & Wolf, 2009; Hertler et al., 2018; Wilson, 1975). Accordingly, organisms trade-off their limited investment into important adaptive tasks such as parenting and mating efforts based on environmental contingencies (Del Giudice et al., 2016; Figueredo et al., 2009). How people calibrate their solutions to mating problems may be facilitated by mate desirability, which allows them to specialize in specific approaches to relationships (Csajbók et al., 2019). Thus, short- and long-term mate value can correlate with short- and long-term mating efforts as a form of optimized mating strategy, while assuming that this may be more nuanced between the sexes. We hypothesize that people who are more focused on parenting efforts should view themselves as having more desirability as a long-term mate given the centrality of these relationships in creating offspring in modern and ancestral environments. In contrast, those who have high short-term desirability will have a more mating-focused life history strategy.

In addition to trade-offs in mating and parenting effort based on life history theory, personality traits are likely to be correlated with self-perceived mate desirability. Specifically, we examine the role of the Dark Triad traits of narcissism (i.e., a sense of grandiosity and egoism), Machiavellianism (i.e., manipulative behaviors and the exploitation of others), and psychopathy (i.e., cruel and callous attitudes and a lack of remorse) to further understand mate desirability. While these traits are reliably and moderately-to-highly correlated with each other (Muris et al., 2017), they may each provide unique insights into individual differences in mate desirability. The traits play a role in mating psychology, but it tends to be confined to short-term mating contexts (Borráz-León & Rantala, 2021; Schmitt et al., 2017; Valentova et al., 2020). If this is the case, we would expect that the Dark Triad traits would be associated with self-perceptions of short-term, but not long-term desirability.

To summarize, evolutionary psychologists assume that people calibrate their mating behaviors based on their own sense of desirability in the market. Nevertheless, the concept suffers from a lack of agreement among researchers about what mate value is, and consequently, exhibits substantial heterogeneity in its measurement. We propose to simply ask participants about their self-perceived desirability in the short- and long-term contexts that reflects simple self-ratings of how desirable one is toward their target relationship partners (akin to Edlund & Sagarin, 2014, but in distinctive contexts). We then explore how these are (1) correlated with life history strategies, the Dark Triad traits, age, self-reported mating success, and a peer-based comparison of desirability, and (2) different in men and women, among those in relationships versus those who are single, and across short- versus long-term relationship contexts.

## Method

### Participants and Procedure

The participants were recruited from 41 countries by an international research collaboration as previously reported (Jonason & Luoto, 2021). Each participant completed the questionnaire in English or in their native language. The survey was translated and back translated by the local researchers (Brislin, 1970). The respondents either participated voluntarily or for course credit. The participants gave their informed consent via tickbox to participate in this anonymous, online survey. Link to data and R codes generating figures and splines is provided in the Data availability statement. SPSS was used to run ANOVAs and correlations, Mplus was used to test the multilevel models.

Altogether 4104 people took part in the questionnaire (63% women), but because of incomplete data, 3895 participants (63% women) were relied on for the current study. Thirteen percent of the participants were from North America, 11% from Central and South America, 40% from Western Europe, 8% from Scandinavia, 24% from Central and Eastern Europe, and 4% from Australasia. This subsample of participants were aged between 18 and 69 (*M* = 24.71, SD = 7.45). Ninety percent of the participants were heterosexual and 47% were single.

### Measures

To assess individual differences in mate desirability, we asked participants to report the ease (1 = *extremely difficult*; 7 = *extremely easy*) with which they can find a short-term (i.e., “If you were single, how easy would it be for you to find a short-term mate for romance?” and “If you were single, how easy would it be for you to find a short-term mate for only sex?”) and a long-term mate (i.e., “If you were single, how easy would it be for you to find a potential long-term mate?” and “If you were single, how easy would it be for you to find a long-term relationship potentially leading to marriage?”). These items were subjected to an exploratory factor analysis using principal axis factoring (Kaiser–Meyer–Olkin = .60; Bartlett’s *χ*^*2*^[6] = 5786.17, *p* <  .001) with Promax (i.e., oblique) rotation revealing two dimensions (Eigen_ltm_ = 2.46; Eigen_stm_ = 1.08) accounting for 88.54% of the variance in these four items. We averaged these items to capture individual differences in self-perceived desirability in the short-term (*ρ* =  .55, *p* <  .001) and long-term (*ρ* =  .79, *p* <  .001) contexts. Because 1029 participants did not have full data coverage on these items, their response was taken from only one item. We chose this approach instead of excluding 26% of the sample, because the subsample having full data coverage in short- and long-term mate desirability had virtually the same descriptive statistics as the large sample (short-term mate desirability: full data coverage *N* = 3,599, *M* = 4.45, SD = 1.50, total sample *N* = 3,895, *M* = 4.47, SD = 1.52; *t*[7492] =  −  .57, *p* >  .05, Cohen’s *d* =  −  .01; long-term mate desirability: full data coverage *N* = 3,162, *M* = 3.01, SD = 1.33, total sample *N* = 3895, *M* = 2.92, SD = 1.33; *t*[7055] = 2.83, *p* <  .01, *d* =  .07).

Individual differences in the Dark Triad traits were measured with the Dirty Dozen (Jonason & Webster, 2010) scale that is composed of 12 items, four each for psychopathy (e.g., “I tend to be unconcerned with the morality of my actions.”), Machiavellianism (e.g., “I have used deceit or lied to get my way.”), and narcissism (e.g., “I tend to want others to pay attention to me.”). Participants reported their agreement with each statement (1 = *strongly disagree*; 5 = *strongly agree*). Items on the respective scales were summed to create indexes of psychopathy (Cronbach’s *α* =  .75), Machiavellianism (*α* =  .67), and narcissism (*α* =  .79).

We also used the Brief Life History Scale (Kruger, 2017), which is an eight-item tool. Four items measured parenting (*α* =  .62; e.g., “Good at taking care of children”) and four measured mating effort (*α* =  .67; e.g., “Sleep with a large number of people in your lifetime”). Participants reported how much (1 = *not at all*; 7 = *very much*) each item described them. The sum of the items of parenting effort, and separately, mating effort, were used as our variables.

And last, we included several single-item measures, which should be reasonably reliable (Dollinger & Malmquist, 2009). We asked participants to “rate how physically attractive you consider yourself” (1 = *very unattractive*; 7 = *very attractive*). We assessed short-term and long-term mating success by asking them to report how many partners they have had for short- versus long-term relationships. These were positively skewed (short-term: *M* = 1.87, Median = 3; *SD* = 9.31, Range = 0–200; Skew = 8.41; Kurtosis = 114.03; long-term: *M* = 5.32, Median = 2; SD = 1.35, Range = 0–30; Skew = 3.36; Kurtosis = 52.33) because the response was zero-loaded (i.e., no charge was posed on a potential exaggeration). Therefore, we took the natural log of both items (after adding one to each because the log function is meaningless at zero) and used them as context-specific measures of mating success. And last, we asked participants to report their short- and long-term mating success relative to their peers (i.e., “In comparison with your peers, who are around the same age as you, would you consider yourself”; 1 = *below average*; 2 = *average*; 3 = *above average*). The two items were correlated (*ρ* =  .38, *p* <  .01) and thus summed to create a measure of peer comparison.

## Results

**Fig. 1:**
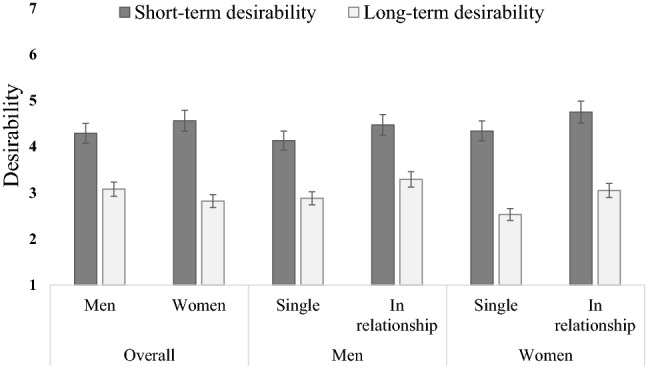
Mean self-perceived short- and long-term desirability ratings across sex and relationship status (5% error bars)

First, we ran a mixed model ANOVA with sex (men/women) and relationship status (single/coupled) as between-subjects variables and context (short-term/long-term) as within-subjects variable on desirability (Fig. 1) and found an interaction of context and sex (*F*[1, 3891] = 109.49, *p* <  .01, *η*_*p*_^*2*^ <  .03), suggesting women felt they had more short-term desirability than men, whereas men felt they had more long-term desirability than women. We also found that participants felt they had more (*F*[1, 3891] = 3268.78, *p* <  .01, *η*_*p*_^*2*^ <  .50) short-term (*M* = 4.47, SD = 1.52) than long-term (*M* = 2.92, SD = 1.33) desirability. And last, we found that people in relationships (*M* = 3.89, SD = 1.14) felt they were more (*F*[1, 3891] = 113.86, *p* <  .01, *η*_*p*_^*2*^ <  .03) desirable than those who were single (*M* = 3.47, SD = 1.21). When we controlled for age as a covariate, although the size of the effect for context (*ΔF* = −3046.75, *Δη*_*p*_^*2*^ = − .40) and relationship status (*ΔF* =  − 11.27, *Δη*_*p*_^*2*^ <  .01) shrunk considerably, the interaction effect of context and sex slightly increased (*ΔF* = 3.01, *Δη*_*p*_^*2*^ <  .01), and the context and relationship status interaction became significant albeit with a trivial effect size given the magnitude of these data (*F*[1, 3891] = 4.58, *p* =  .03, *η*_*p*_^*2*^ <  .01). According to this interaction, short-term desirability is higher than long-term desirability, and this difference is more articulated in single (estimated *ΔM* = 1.54) than in coupled (estimated *ΔM* = 1.42) participants (although single participants rated their desirability lower than the coupled participants).

Second, we assessed how self-rated mating desirability varies with age, self-perceived attractiveness, peer comparison, mating success, life history strategy, and the Dark Triad traits (Table 2). Short- and long-term desirability self-ratings were correlated overall (*r* =  .40, *p* <  .01), in men (*r* =  .45, *p* <  .01), and in women (*r* =  .38, *p* <  .01); the correlation was larger in men than in women (Fisher’s *z* = 2.54, *p* <  .05). Self-perceived short-term desirability correlated more strongly (in positive direction) with self-perceived physical attractiveness, peer comparison, short-and long-term mating success, mating oriented life history strategy, Machiavellianism, psychopathy, and narcissism than self-perceived long-term desirability in the overall sample. This pattern of correlations was the same for both sexes. Self-perceived long-term desirability correlated more strongly (in positive direction) with parenting life history strategy than short-term desirability in the overall sample, for both men and women. However, we also found stronger correlations between short- and long-term self-perceived desirability and age in men (positive) than in women (non-significant). Also, there were stronger positive correlations between the number of long-term partners, Machiavellianism, and short-term desirability in men than in women. Long-term desirability more strongly and positively correlated with mating life history in men than in women. In contrast, we found stronger positive correlations between the number of short-term partners and long-term desirability, and between narcissism and short-term desirability in women than in men. In addition, self-perceived attractiveness did not correlate with age in men (*r* =  −  .04, *p* >  .05), but weakly and positively correlated in women (*r* =  .07, *p* <  .01).

**Table 2:** Correlations between short- and long-term desirability and age, self-perceived physical attractiveness, life history strategies, and the Dark Triad traits overall, across mating context (Steiger’s *z*), and when compared in men and women within each context (Fisher’s *z*)

	Overall			Men			Women			Sex differences	
	STD	LTD	*z*	STD	LTD	*z*	STD	LTD	*z*	STD	LTD
Age	.05**	.05**	< 0.01	.10**	.12**	− .72	− .03	−.02	− .45	3.92**	4.23**
Physical attractiveness	.50**	.27**	14.73**	.52**	.31**	4.98**	.48**	.26**	10.96**	1.60	1.64
Peer comparison	.18**	.06**	6.89**	.21**	.08**	8.65**	.16**	.04*	5.40**	1.56	1.21
*N* of short-term partners	.33**	.09**	14.18**	.37**	.13**	9.10**	.33**	.03	13.90**	1.37	3.03**
*N* of long-term partners	.22**	.17**	2.91**	.27**	.20**	2.61**	.19**	.15**	1.82	2.54**	1.55
Life History—parenting	< .01	.12**	−6.78**	< .01	.13**	−4.44**	−.02	.14**	−7.00**	0.63	1.94
Life History—mating	.35**	.09**	15.14**	.41**	.12**	11.12**	.36**	.04*	14.75	−0.37	2.36*
Machiavellianism	.13**	< .01	7.42**	.18**	.01	6.17**	.12**	−.02	6.27**	1.85*	0.90
Psychopathy	.04*	−.03*	3.97**	.06*	−.07**	4.68**	.07**	−.05*	5.37**	−0.30	−0.60
Narcissism	.11**	.03	4.56**	.08**	.03	1.80	.14**	.02	5.39**	−1.83*	0.30

*STD* short-term desirability; *LTD* long-term desirability

* *p* < .05, ** *p* < .01

**Fig. 2:**
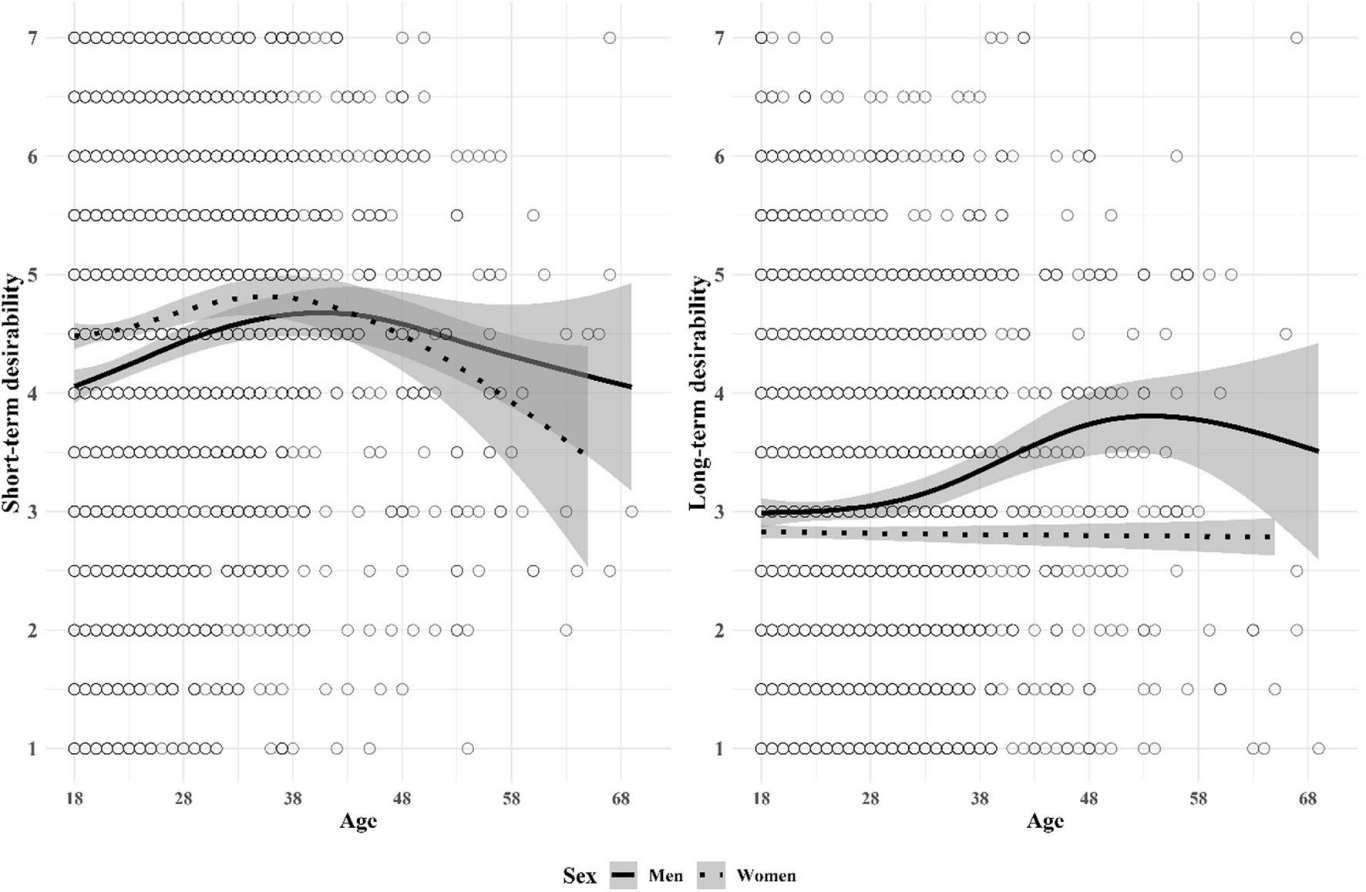
Smoothing splines fitted on the association between age and short- and long-term desirability across sex (95% confidence interval)

**Fig. 3:**
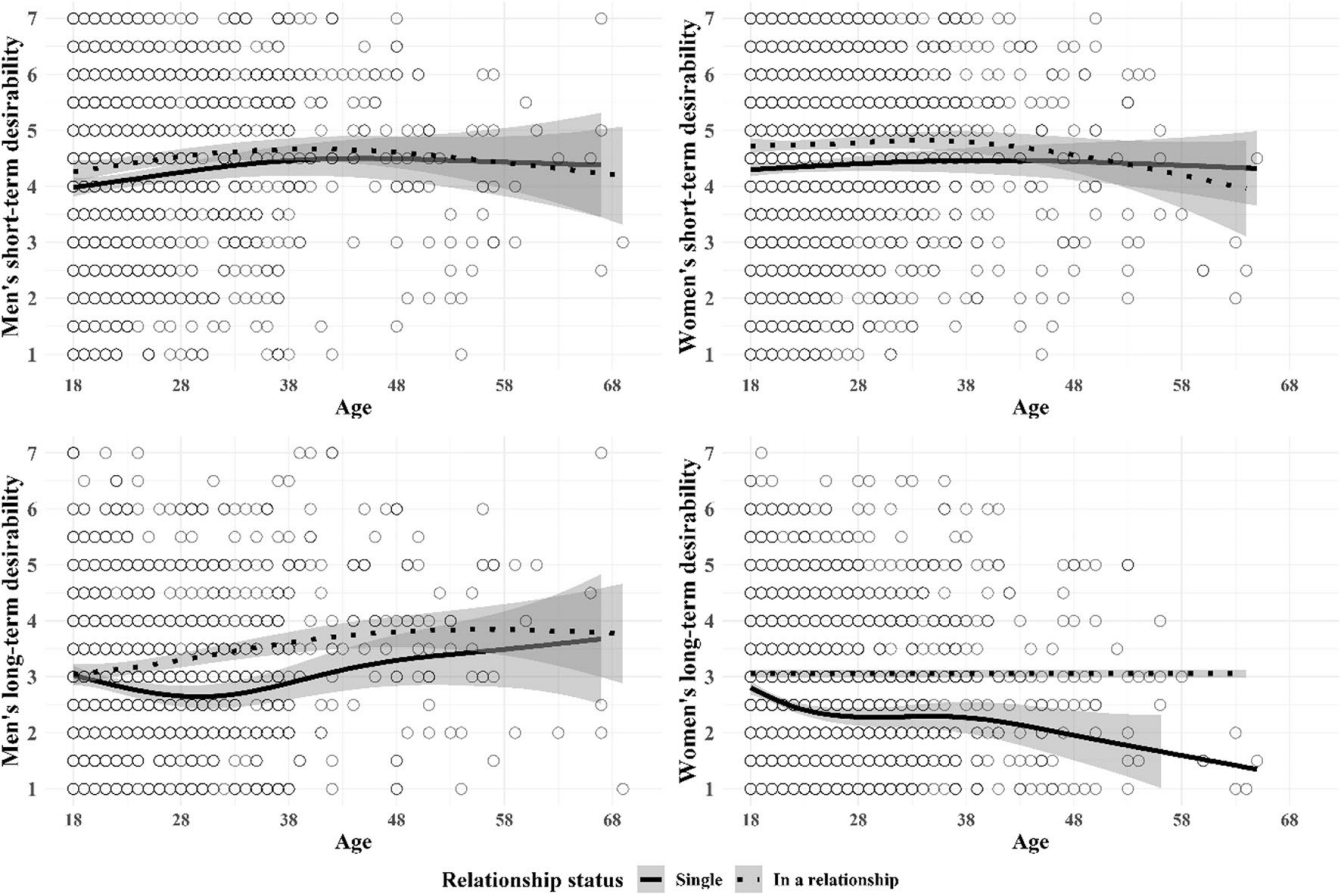
Smoothing splines fitted on the association between age and short- and long-term desirability in men and women across relationship status (95% confidence interval)

Third, to better understand the nonlinear nature of mating desirability over the life course, we plotted the associations between age and short- and long-term desirability in men and women and relationship status using smoothing splines to explore the shape of the association without any constraints. The formula for the regression spline used the Generalized Additive Model (GAM) defined by piecewise cubic terms, shrinkage, and four knots (geom_smooth function in ggplot2 R package; James et al., 2013). The regression splines (Figs. 2 and 3), defined on the association between age and the desirability ratings, showed that (1) men’s self-perceived short-term desirability increased up to the age of 40, and slightly decreased afterwards whereas women’s self-perceived short-term desirability slightly increased with a less steep slope than men’s up to the age of 38 but decreased afterwards with a steeper velocity than men’s desirability, (2) men’s long-term desirability increased up to the age of 50 and decreased afterwards whereas women’s long-term desirability was stable over time, (3) single women’s short-term desirability decreased until the age of 30 but increased afterwards, (4) women in relationships had an increasing self-perceived short-term desirability over time, (5) long-term desirability decreased among coupled men after the age of 38 and remained stable among single men, and last (6) coupled women’s long-term desirability was stable across age and steeply decreased among single women. The cubic versus spline regression results are reported in Table 3.

**Table 3:** Associations between short- and long-term desirability and age using cubic and splines regression methods across sex, context, and relationship status

Sex	Context	Relationship status	Cubic		Spline	
			*F*	*R*^*2*^	*F*	*R*^*2*^
Men	Short	Pooled	13.25**	.02	8.84**	.02
		Single	4.33*	.01	2.93*	.01
		Coupled	5.05**	.01	3.39*	.01
	Long	Pooled	10.79**	.01	10.95**	.02
		Single	4.08*	.01	4.26**	.02
		Coupled	10.50**	.03	9.10**	.04
Women	Short	Pooled	8.03**	< .01	6.46**	< .01
		Single	2.05	< .01	2.21	< .01
		Coupled	2.66	< .01	2.98*	< .01
	Long	Pooled	0.71	< .01	4.07**	< .01
		Single	14.25**	.03	12.11**	.03
		Coupled	0.08	< .01	1.61	< .01

* *p* < .05, ** *p* < .01

Fourth, because age had a skewed distribution (*M* = 24.71, SD = 7.45, Median = 22, Skewness = 2.10, Kurtosis = 5.44) and our sample underrepresented participants over 30 years of age, we created age-groups to test the sensitivity of the regression splines. Table 4 contains the short- and long-term desirability ratings across age categories. Just like in the main analysis, context had a main effect (*F*[1, 3881] = 1062.95, *p* <  .01, *η*_*p*_^*2*^ =  .22) and interacted with participant’s sex (*F*[1, 3881] = 49.87, *p* <  .01, *η*_*p*_^*2*^ =  .01). Context and age interacted (*F*[6, 3881] = 7.53, *p* <  .01, *η*_*p*_^*2*^ =  .01), as well as participant’s sex and age (*F*[6, 3881] = 3.31, *p* <  .01, *η*_*p*_^*2*^ =  .01). Participant’s sex (*F*[1, 3881] = 5.79, *p* =  .02, *η*_*p*_^*2*^ <  .01) and age-groupings had a main effect (*F*[6, 3881] = 7.19, *p* <  .01, *η*_*p*_^*2*^ =  .01). Bonferroni post hoc tests suggested that men aged between 18 and 25 had the lowest self-perceived short-term desirability, while men aged between 41 and 50 years had the highest short-term desirability. Women’s short-term desirability was the highest between their age of 26–30, but the lowest mate desirability group between the age of 51–69 was not confirmed by tests. Men’s self-perceived long-term desirability was highest again between the age of 41–50, with no clear lowest rating group. Women’s long-term desirability did not show significant age-category patterns (similarly to the spline results).

**Table 4:** Mean short- and long-term desirability across age groups

		STD		LTD	
	*N*	Mean	SD	Mean	SD
*Overall*					
18–20 years	1276	4.39	1.54	2.96	1.31
21–25 years	1442	4.37	1.52	2.82	1.28
26–30 years	539	4.70	1.48	2.87	1.30
31–35 years	299	4.65	1.41	2.93	1.33
36–40 years	164	4.80	1.60	3.27	1.59
41–50 years	117	4.76	1.50	3.26	1.54
51–69 years	58	3.90	1.34	3.17	1.44
*Men*					
18–20 years	420	4.15	1.57	3.08	1.40
21–25 years	481	4.13	1.52	2.93	1.37
26–30 years	221	4.52	1.48	2.96	1.37
31–35 years	130	4.57	1.45	3.19	1.36
36–40 years	87	4.66	1.69	3.40	1.68
41–50 years	54	4.95	1.40	3.90	1.52
51–69 years	37	3.92	1.33	3.50	1.41
*Women*					
18–20 years	856	4.51	1.52	2.90	1.26
21–25 years	961	4.49	1.51	2.76	1.22
26–30 years	318	4.82	1.46	2.81	1.26
31–35 years	169	4.70	1.39	2.73	1.28
36–40 years	77	4.97	1.48	3.11	1.49
41–50 years	63	4.60	1.58	2.71	1.35
51–69 years	21	3.86	1.39	2.60	1.33

*SD* standard deviation, *STD* short-term desirability, *LTD* long-term desirability

Fifth, we conducted multilevel modeling to study how country origins affected the effects of sex, context, age, and relationship status on desirability. To avoid biased regression estimates, here we excluded 63 participants whose country was underrepresented (i.e., sample size below 50 within a country; Maas & Hox, 2005). We thus used 13 countries which functioned as clustering variables predicting the random intercepts of desirability ratings. In the unconditional Model 0, we separated the variance of desirability into within country and between country variances where the intraclass correlation (ICC) was equal to 0.03 indicating low between country variability. Entering context (short vs long), sex, and their interaction as fixed effects in Model 1 explained almost 25% of the within country variance (Table 5, Supplementary Table 1). Adding age to Model 2 increased the explained variance by 0.31%. Note that age-squared and relationship status could not be accounted for in a shared model because of the relatively low number of clusters. When using relationship status instead of age, the overall model explained almost 27% of the within country variance (Table 6, Supplementary Table 2). All predicting effects of sex, context, relationship status, age, and their interactions were in accordance with the results of ANOVA that unaccounted for country clusters.

**Table 5:** Multilevel models predicting self-perceived desirability with sex, context, and age, adjusting for country of origin

	Predictors (fixed effects)^a^	Desirability
ICC (Model 0)		.03
Model 1	Context (short)	1.75**
	Sex (men)	0.14**
	Sex × Context	−0.53**
Explained variance^b^		24.73%**
Model 2	Context (short)	1.50**
	Sex (men)	−0.37*
	Age	−0.01
	Sex × Context	−0.25
	Sex × Age	0.02**
	Context × Age	0.01
	Sex × Context × Age	−0.01
Explained variance^c^		0.31%**

*ICC* intraclass correlation, that is variance attributable to cluster (country) differences

^a^The models are clustered across countries

^b^Explained within country residual variance of the fixed effect of sex and context in comparison with Model 0 (i.e., unconditional model)

^c^Explained within country residual variance of the fixed effect of sex, context, and age in comparison with Model 1

*p < .05. **p < .01

**Table 6:** Multilevel models predicting self-perceived desirability with sex, context, and relationship status, adjusting for country of origin

	Predictors (fixed effects)^a^	Desirability
ICC (Model 0)		.03
Model 1	Context (short)	1.83**
	Sex (men)	0.23*
	Relationship (yes)	0.56**
	Sex × Context	−0.57**
	Sex × Relationship	−0.11
	Context × Relationship	−0.13
	Sex × Context × Relationship	0.06
Explained variance^b^		26.87%**

*ICC* intraclass correlation, that is variance attributable to cluster (country) differences

^a^The models are clustered across countries

^b^Explained within country residual variance of the fixed effect of sex, context, and relationship status in comparison with Model 0 (i.e., unconditional model)

*p < .01, ** *p* < .001

Sixth, although we did not predict any country differences, for descriptive purposes we tested the country effect on short- and long-term desirability excluding countries having sample sizes below 100 participants (Supplementary Table 3). Context had a main effect in this subsample as well (*F*[1, 3645] = 2547.32, *p* <  .01, *η*_*p*_^*2*^ =  .41) and interacted with participants’ sex (*F*[1, 3645] = 75.56, *p* <  .01, *η*_*p*_^*2*^ =  .02), country (*F*[10, 3645] = 13.20, *p* <  .01, *η*_*p*_^*2*^ =  .04), as well as sex and country (*F*[10, 3645] = 2.10, *p* =  .02, *η*_*p*_^*2*^ =  .01). Sex had a main effect (*F*[1, 3645] = 6.01, *p* =  .01, *η*_*p*_^*2*^ <  .01), just like country (*F*[10, 3645] = 29.30, *p* <  .01, *η*_*p*_^*2*^ =  .07), and they interacted (*F*[10, 3645] = 2.39, *p* <  .01, *η*_*p*_^*2*^ =  .01). Post hoc* t-*tests revealed that Brazilian men had more long-term desirability than Brazilian women did (*t*[199.13] =  − 2.72, *p* <  .01) just like British (*t*[261.99] =  − 1.94, *p* =  .05) and American men (*t*[283] =  − 2.35, *p* =  .02). Canadian women had more short-term desirability than Canadian men did (*t*[211] = 2.55, *p* =  .01) just like Czech (*t*[727] = 5.17, *p* <  .01), Danish (*t*[194.91] = 6.01, *p* <  .01), Dutch (*t*[347] = 2.38, *p* =  .02), Romanian (*t*[201] = 1.93, *p* =  .06), and American women (*t*[283] = 2.49, *p* =  .01).

## Discussion

Mate value is a complex and widely used concept, however, its operationalization is still ambiguous. Mate value comprises objectively valued mating qualities (e.g., physical attractiveness; Csajbók & Berkics, 2017; Singh, 2002), individually valued qualities (e.g., the same level of education; Luo, 2017; Štěrbová & Valentova, 2012), and self-perceptions (e.g., self-esteem; Brase & Guy, 2004; Csajbók et al., 2019; Goodwin et al., 2012; Surbey & Brice, 2007). Moreover, the inter-individual agreement on one’s mate value may vary depending on several factors (e.g., mating context). Nevertheless, mate value is an important predictor of human mating behavior (Arnocky, 2018). Here, we created a simple measure of it using self-perceived mate desirability (based on direct questions about how desirable one feels) to investigate its predicted correlates stemming from mate value research. Our results are in line with theories on mating market operations. In accord with previous studies, short- and long-term mate desirability correlate in an expected way with relationship status, the Dark Triad traits, life history strategies, peer-based comparison of desirability, and mating success. In sum, measuring long- and short-term self-perceived desirability can be used in future research, especially when a brief measure would be particularly useful.

In more detail, short-term desirability correlated more strongly with self-perceived physical attractiveness, peer comparison, mating success, mating life history strategy, Machiavellianism, psychopathy, and narcissism than long-term desirability in both sexes. Otherwise, long-term desirability correlated more strongly with parenting life history strategy than short-term desirability. These results are in line with previous research (Borráz-León & Rantala, 2021; Buss & Schmitt, 1993; Csajbók & Berkics, 2017; Csajbók et al., 2019; Valentova et al., 2020). However, so far there is only limited research (and no psychometrics work) with a mate value scale differentiating short- and long-term self-perceived desirability (Jonason et al., 2019, 2020), and thus we observed some differences in comparison with results obtained with general mate value measures.

Previous research either did not find sex differences in general mate value (Brase & Guy, 2004; Goodwin et al., 2012; Mafra & Lopes, 2014), or found that women had higher self-perceived mate value than men when measuring mate value without differentiating the mating context (Csajbók et al., 2019). Here, mate desirability was sex- and context-dependent with men reporting more overall desirability than women. Men felt they had more long-term desirability, while women felt they had more short-term desirability. We posit that this sex difference reflects the relationship (i.e., strong correlation) between short-term mate desirability and physical appearance, which is especially important in women’s mating strategies. Also, women usually have more short-term mating offers than men (Timmermans & Courtois, 2018), and thus it is no surprise that their self-perceived short-term desirability is also higher in accordance with the sexual strategies theory (Buss & Schmitt, 1993). In contrast, long-term mate desirability was correlated with age in men, but not in women, and therefore it might have functioned in men as a combination of shifting their efforts from short-term mating to long-term mating, good parenting, and accruing resource capacities (Brase & Guy, 2004; Buss & Schmitt, 1993). However, why men had more overall desirability (irrespective of the context) than women might be a function of either their greater tendency to be narcissistic (Grijalva et al., 2015) or women’s greater tendency to have negative views of themselves in physical appearance (Zeigler-Hill & Myers, 2012), which might not reflect how others perceive their attractiveness (Pereira et al., 2019).

An individual’s self-perceived desirability is weakly dependent on age (Csajbók et al., 2019). Our results indicate that age-related trajectories of mate desirability vary in men and women and mating contexts. In women, short-term desirability increases up to age 38 and decreases afterward, which is in concordance with evolutionary explanations highlighting the importance of youth and fertility in women attracting men (Fisher, 2004; Pawlowski & Dunbar, 1999; Singh, 2002). Interestingly, women’s long-term desirability remains the same from ages 18 until 68. Importantly, women’s self-perceived attractiveness, which otherwise strongly correlated with short-term desirability, more strongly correlated with age (and in a positive direction) than short-term desirability. These findings may reflect that in long-term relationships, women’s valued qualities (e.g., emotional stability) are not as dependent on age as qualities signaling desirability for short-term relationships (e.g., physical attractiveness; Csajbók & Berkics, 2017; Li & Kenrick, 2006). On the other hand, the qualities might partly compensate for each other (e.g., decreasing physical attractiveness after the age of 38 might be compensated by increasing parental skills).

In men, short-term desirability shows a similar pattern with age as in women (the peak was around 40 years, but the decrease was less steep). The peak of short-term desirability might be interpreted by higher socio-economic status which rises with age but is then opposed by decreasing sexual performance (Brase & Guy, 2004; Mafra & Lopes, 2014; but see Csajbók et al., 2019). Interestingly, the pattern of long-term desirability by age shows more variation than the pattern of short-term desirability by age between the sexes. In men, long-term desirability slowly increases until 50 years, and then slowly decreases, while no change is seen among women. Therefore, we found that age-dependent trajectories of desirability differ by sex and mating context. However, as mentioned above, these age associations were weak. Possibly because, for example, a 60-year-old woman may be imagining how 60–70 years old men perceive her, not how a 25-year-old man perceives her. There may be many differences in reference frames depending on the age of participants, which supports our reasoning that mate value is subjective (cf. social comparison, Festinger, 1954).

People form couples based on self-similarity in mate value (Conroy-Beam et al., 2019b; Ellis & Kelley, 1999). If the distribution of mate desirability is normal, it might be more demanding for individuals with high mate desirability to find a partner with self-similar mate desirability, even though more desirable individuals have, on average, more mating offers (Maestripieri et al., 2017). Individuals with more mate value have higher mating standards, therefore they also reject more mating offers than less desirable individuals (Csajbók et al., 2019; Jonason et al., 2015; Wenzel & Emerson, 2009). From this perspective, their mating market is, in reality, more limited than one might expect. However, mate desirability affects short- and long-term mating success in a different way. First, self-similarity in most characteristics is universal and higher in long-term couples (Conroy-Beam et al, 2019b; Felmlee, 2001; Štěrbová & Valentova, 2012), and second, the level of choosiness is also higher in the long-term mating context (Csajbók & Berkics, 2017, 2022; Fletcher et al., 2004). From this perspective, individuals who are especially desirable as a mate might be more successful in both mating contexts than those with low mate desirability, albeit the mate choice processes can be different.

### Limitations and Conclusions

Despite the large sample size, cross-cultural data, and straight-forward measurement properties, our study had several shortcomings. First, with our reliance on college-student participants, our sample was richer, more democratic, industrialized, and more educated than much of the world (Heinrich et al., 2010; Rad et al., 2018). Second, our measurement of mate value is based on self-reports of desirability. We lack any external referencing points to validate these self-ratings. Nevertheless, with our face-valid approach, we think this way of conceptualizing mate value as mate desirability is promising, and potentially more easily interpreted. As this current research was part of a large international study, here we could not correlate our new measure with already existing convergent and discriminant measures of mate value and related constructs. In the future, more studies will be needed also to test various ways this measure may or may not be valid. On a related note, when we used metrics composed of two items, they had only modest correlations between them as a test for their internal consistency. Item-level correlations are generally lower than multi-item metrics like Cronbach’s α used for these purposes (Eisinga et al., 2013). Third, while we examined some cross-national variance, we did not attempt to account for this because (1) we had no predictions about such effects and (2) the sample size of nations is likely too low to detect country-level correlations. Future research might consider the role of ecological contingencies that allow for mate value calibration, including but not limited to experimental effects (e.g., bogus feedback studies) and nation-level correlates (in a substantially larger sample) with factors like the operational sex ratio (e.g., women having higher self-perceived mate desirability in a society with more numerous men and vice versa; see Walter et al., 2021).

In conclusion, we have attempted to better understand and simplify the concept of mate value. We argue that prior assessments were inconsistent and limited (e.g., focused on self-evaluations of specific qualities, failure to consider context effects) and that objective estimates of mate value are untenable, therefore face-valid assessments of one’s self-perceived desirability can be informative. We employed an ad hoc, bidimensional self-report measure of desirability and explored sex differences, context effects, relationships status differences, and associations with age (linear and curvilinear), the Dark Triad traits, self-rated physical attractiveness, mating success, and relative desirability, and two aspects of life history strategies. As predicted, we found that objective indicators such as age and sex affected mate value in the expected directions, but these effects were relatively weak. Instead, self-perceived physical attractiveness and mating strategy were more strongly associated with self-perceived mate value. We suggest that more relativistic, dynamic, and transactional perspectives such as social comparison, the sociometer model, and theories on market operations better explain self-perceived mate value than objective metrics like facial symmetry, waist-to-hip ratio, fecundity, or grip strength.

## Supplementary Information

Below is the link to the electronic supplementary material.

Supplementary file1 (DOCX 25 KB)

## Data Availability

Data and R-code for this study are available on the Open Science Framework: https://osf.io/shgzj/?view_only=0fea3d18ee49463c8aef93f10157f85d. This study was not preregistered.
