# Supplementary material for: Individual Differences in How Desirable People Think They Are as a Mate
Source: Arch Sex Behav. 2023 May 8;52(6):2475–90. doi: 10.1007/s10508-023-02601-x (PMC10501943; doi:10.1007/s10508-023-02601-x)
Supplement: Supplementary file 1 — Supplementary file1 (DOCX 25 KB) [file 10508_2023_2601_MOESM1_ESM.docx]

| **Supplementary Table 1.**  Detailed results of the multilevel models predicting self-perceived desirability with sex, context, and age, adjusting for country of origin | | |
| --- | --- | --- |
| **Model** |  | **Desirability** |
| Unconditional model (Model 0) | Unexplained within country variance | 2.53** |
|  | Unexplained between country variance | .08* |
|  | Log-likelihood | -14450.89 |
|  | *N* of free parameters | 3 |
| Fixed effects of sex and context (Model 1) | Unexplained within country variance | 1.91** |
|  | Unexplained between country variance | .09* |
|  | Log-likelihood | -13365.02 |
|  | *N* of free parameters | 6 |
| Comparison of Model 1 with Model 0 | Change in deviance, change in df | 2171.73(3)** |
|  | Added explained within country residual variance (compared to Model 0) | 24.73%** |
| Fixed effects of sex, context, and age (Model 2) | Unexplained within country variance | 1.90** |
|  | Unexplained between country variance | .08* |
|  | Log-likelihood | -13352.70 |
|  | *N* of free parameters | 10 |
| Comparison of Model 2 with Model 1 | Change in deviance, change in df | 24.64(4)** |
|  | Added explained within country residual variance (compared to Model 1) | .31%** |
| *Note*. df = degrees of freedom  * *p* < .05*.* ** *p* < .01 | | |

| **Supplementary Table 2.**  Detailed results of the multilevel models predicting self-perceived desirability with sex, context, and relationship status, adjusting for country of origin | | |
| --- | --- | --- |
| **Model** |  | **Desirability** |
| Unconditional model (Model 0) | Unexplained within country variance | 2.53** |
|  | Unexplained between country variance | .08* |
|  | Log-likelihood | -14450.89 |
|  | *N* of free parameters | 3 |
| Fixed effects of sex, context, and relationship (Model 1) | Unexplained within country variance | 1.85** |
|  | Unexplained between country variance | .09* |
|  | Log-likelihood | -13255.38 |
|  | *N* of free parameters | 10 |
| Comparison of Model 1 with Model 0 | Change in deviance, change in df | 2391.02(7)** |
|  | Added explained within country residual variance (compared to Model 0) | 26.87%** |
| *Note*. df = degrees of freedom  * *p* < .05, ** *p* < .01 | | |

| **Supplementary Table 3.**  Country differences in short- and long-term desirability across sex | | | | | | |
| --- | --- | --- | --- | --- | --- | --- |
|  |  |  | Short-term desirability | | Long-term desirability | |
| Sex | Country | *N* | Mean | *SD* | Mean | *SD* |
| Women | Australia | 84 | 4.56 | 1.59 | 3.46 | 1.36 |
|  | Belgium | 482 | 3.72 | 1.21 | 2.51 | 1.20 |
|  | Brazil | 214 | 4.58 | 1.44 | 2.48 | 1.24 |
|  | Canada | 153 | 5.33 | 1.26 | 3.31 | 1.37 |
|  | Czech | 543 | 4.58 | 1.48 | 2.65 | 1.07 |
|  | Denmark | 84 | 5.71 | 1.26 | 3.36 | 1.50 |
|  | Germany | 125 | 4.86 | 1.38 | 2.71 | 1.05 |
|  | Netherland | 234 | 4.74 | 1.61 | 3.04 | 1.19 |
|  | Romania | 114 | 5.12 | 1.44 | 2.90 | 1.38 |
|  | United Kingdom | 158 | 4.35 | 1.47 | 3.01 | 1.19 |
|  | USA | 143 | 4.82 | 1.53 | 2.98 | 1.43 |
|  | Total | 2334 | 4.55 | 1.50 | 2.80 | 1.25 |
| Men | Australia | 37 | 4.26 | 1.72 | 3.42 | 1.61 |
|  | Belgium | 189 | 3.63 | 1.20 | 2.62 | 1.37 |
|  | Brazil | 116 | 4.37 | 1.55 | 2.92 | 1.51 |
|  | Canada | 60 | 4.81 | 1.55 | 3.43 | 1.44 |
|  | Czech | 186 | 3.93 | 1.48 | 2.62 | 1.10 |
|  | Denmark | 219 | 4.66 | 1.64 | 3.36 | 1.52 |
|  | Germany | 43 | 4.94 | 1.10 | 2.99 | 1.36 |
|  | Netherland | 115 | 4.29 | 1.76 | 3.00 | 1.26 |
|  | Romania | 89 | 4.75 | 1.29 | 3.04 | 1.37 |
|  | United Kingdom | 137 | 4.20 | 1.60 | 3.32 | 1.47 |
|  | USA | 142 | 4.38 | 1.49 | 3.37 | 1.38 |
|  | Total | 1333 | 4.29 | 1.55 | 3.05 | 1.42 |
| Overall | Australia | 121 | 4.47 | 1.63 | 3.45 | 1.43 |
|  | Belgium | 671 | 3.70 | 1.20 | 2.54 | 1.25 |
|  | Brazil | 330 | 4.51 | 1.48 | 2.63 | 1.36 |
|  | Canada | 213 | 5.19 | 1.37 | 3.35 | 1.39 |
|  | Czech | 729 | 4.41 | 1.51 | 2.64 | 1.08 |
|  | Denmark | 303 | 4.95 | 1.61 | 3.36 | 1.51 |
|  | Germany | 168 | 4.88 | 1.31 | 2.78 | 1.14 |
|  | Netherland | 349 | 4.59 | 1.67 | 3.03 | 1.21 |
|  | Romania | 203 | 4.96 | 1.39 | 2.96 | 1.37 |
|  | United Kingdom | 295 | 4.28 | 1.53 | 3.15 | 1.33 |
|  | USA | 285 | 4.60 | 1.52 | 3.18 | 1.42 |
|  | Total | 3667 | 4.45 | 1.52 | 2.89 | 1.32 |
| *Note. SD* = standard deviation. | | | | | | |
